# Dysfunction of ATG7-dependent autophagy dysregulates the antioxidant response and contributes to oxidative stress-induced biological impairments in human epidermal melanocytes

**DOI:** 10.1038/s41420-020-0266-3

**Published:** 2020-05-01

**Authors:** Zhuhui Qiao, Zhongyi Xu, Qing Xiao, Yiwen Yang, Jiayi Ying, Leihong Xiang, Chengfeng Zhang

**Affiliations:** Department of Dermatology, Huashan Hospital, Fudan University, Shanghai, China

**Keywords:** Macroautophagy, Vitiligo

## Abstract

Autophagy is a process involving the self-digestion of components that participates in anti-oxidative stress responses and protects cells against oxidative damage. However, the role of autophagy in the anti-oxidative stress responses of melanocytes remains unclear. To investigate the role of autophagy in human epidermal melanocytes, we knocked down and overexpressed ATG7, the critical gene of autophagy, in normal human epidermal melanocytes. We demonstrated that ATG7-dependent autophagy could affect melanin content of melanocytes by regulating melanogenesis. Moreover, suppression of ATG7-dependent autophagy inhibits proliferation and promotes oxidative stress-induced apoptosis of melanocytes, whereas enhancement of ATG7-dependent autophagy protects melanocytes from oxidative stress-induced apoptosis. Meanwhile, deficiency of ATG7-dependent autophagy results in premature senescence of melanocytes under oxidative stress. Notably, we verified that ATG7-dependent autophagy could alter oxidative stress homeostasis by regulating reactive oxygen species (ROS) production, nuclear factor erythroid 2-related factor 2 (Nrf2) antioxidant pathway, and the activity of several antioxidant enzymes in melanocytes. In conclusion, our study suggested that ATG7-dependent autophagy is indispensable for redox homeostasis and the biological functions of melanocytes, such as melanogenesis, proliferation, apoptosis, and senescence, especially under oxidative stress.

## Introduction

Human skin is directly and constantly under the risks of numerous oxidative environmental stressors, such as ultraviolet (UV) irradiation, air pollutants, and chemical products. Redox balance between free radicals and antioxidants, which detoxify their harmful effects is critical for maintaining normal functions of various skin cell types, including keratinocytes, melanocytes, and fibroblast^1–3^. Excessive oxidative stress may lead to skin problems, especially aging^4^ and skin cancer^5^. Recently, oxidative stress has also come to light as a possible mechanism in autoimmune skin diseases^6^, inflammatory skin diseases^7^, and pigmentation skin disorders, such as vitiligo^8–10^. Theoretically, reactive oxygen species (ROS) can attack melanocytes and interfere with normal metabolism, proliferation, and differentiation of melanocytes, which ends up causing cell apoptosis and defects^11^. Studies have indicated that dysfunction of the anti-oxidative system in patients with vitiligo may increase the vulnerability of melanocytes to oxidative damage. Accumulation of ROS will further lead to the impairment of redox homeostasis and result in the destruction of melanocytes^12,13^. However, effective treatment options for vitiligo are still lacking^14^, thus driving researchers to seek potential new therapies.

Autophagy, also known as “self-eating,” is a lysosomal-dependent degradation pathway that is widely present in eukaryotic cells. Under conditions of starvation, infection, or oxidative stress, it can regulate the degradation of long-lived proteins and organelles in cells so that the degradation products can be reused by cells^15^. We previously revealed that autophagy plays a crucial role in the anti-oxidative stress response of skin keratinocytes^1^. Besides, we have confirmed that autophagy functions in both humans and murine melanocytes, and deficiency of ATG7-dependent autophagy can lead to diminished melanin content of dorsal hair, decreased proliferation and premature senescence of melanocytes in mice^2^. However, whether autophagy participates in redox homeostasis and the biological functions of natural human epidermal melanocytes (NHEMs) under oxidative stress remains unknown.

In this study, we aimed to investigate melanogenesis, proliferation, senescence, and oxidative stress response of autophagy-competent and autophagy-deficient NHEM. We showed that ATG7-dependent autophagy had a great influence on melanogenesis, proliferation, apoptosis, and senescence of NHEM, especially under oxidative stress. Meanwhile, it altered oxidative stress homeostasis by regulating reactive ROS production, nuclear factor erythroid 2-related factor 2 (Nrf2) antioxidant pathway, and the activity of several antioxidant enzymes.

## Results

### Knockdown or overexpression of ATG7 efficiently could suppress or enhance autophagy in NHEM

**Fig. 1:**
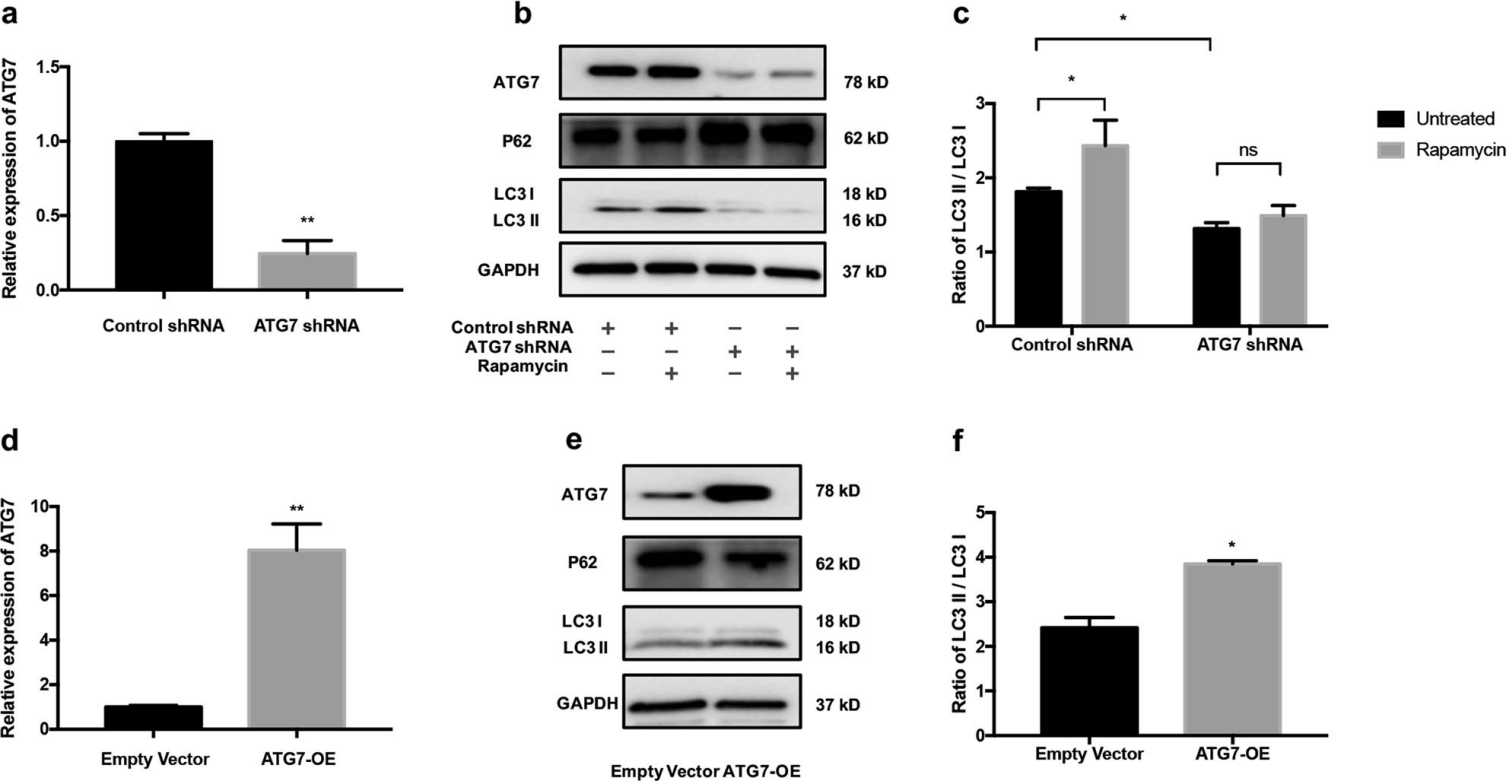
ATG7-dependent autophagy is constitutively suppressed or overexpressed in NHEM. **a** NHEM were transfected with control shRNA or ATG7 shRNA for 3 days. Relative mRNA expression of ATG7 was determined using quantitative reverse transcriptase in real-time PCR (qRT-PCR). **b** Representative images of western blotting of ATG7, p62, and microtubule-associated protein light chain 3 (LC3) in NHEM transfected with control shRNA or ATG7 shRNA in the absence or presence of rapamycin at 40 nM for 36 h. GAPDH was used as a protein loading control. **c** Statistical analysis of western blotting data of ratio of LC3-II and LC3-I in NHEM transfected with control shRNA or ATG7 shRNA in the absence or presence of rapamycin. Data are presented as mean ± SD, *n* = 3 in each group. *P < 0.05, ns, nonsignificant. **d** Relative mRNA expression of ATG7 in NHEM transfected with lentivirus expressing ATG7 or empty vector. **e** Representative images of western blotting of ATG7, p62, and LC3 in NHEM transfected with lentivirus expressing ATG7 or empty vector. GAPDH was used as a protein loading control. **f** Statistical analysis of western blotting data of ratio of LC3-II and LC3-I in NHEM transfected with lentivirus expressing ATG7 or empty vector. Data are presented as mean ± SD, *n* = 3 in each group. *P < 0.05.

To specifically knock down *ATG7* gene in NHEM, the ATG7 short hairpin RNA (shRNA) and control shRNA were transfected. Quantitative reverse transcriptase in real-time PCR (qRT-PCR) analysis showed that the mRNA expression of ATG7 in NHEM was significantly decreased in ATG7 shRNA group (Fig.1a). Western blotting analysis further demonstrated that the protein expression of ATG7 in melanocytes was also diminished in ATG7 shRNA group. Meanwhile, the expression of the autophagy adapter protein p62 was increased and the conversion of LC3-I to LC3-II was decreased in ATG7 shRNA group compared with the control group. Upon rapamycin treatment, conversion ratio of LC3 increased in the control group, whereas the addition of rapamycin caused no increase in LC3-II in the ATG7 shRNA group (Fig. 1b, c). By contrast, ATG7 overexpression by lentivirus transfection significantly enhanced the mRNA and protein levels of ATG7, whereas the expression of p62 was downregulated in NHEM (Fig. 1d, e, f). Collectively, these data indicated that knockdown or overexpression of ATG7 could efficiently suppress or enhance autophagy in NHEM.

### Inactivation of ATG7-dependent autophagy affects melanin content and leads to dysfunction of melanogenesis

**Fig. 2:**
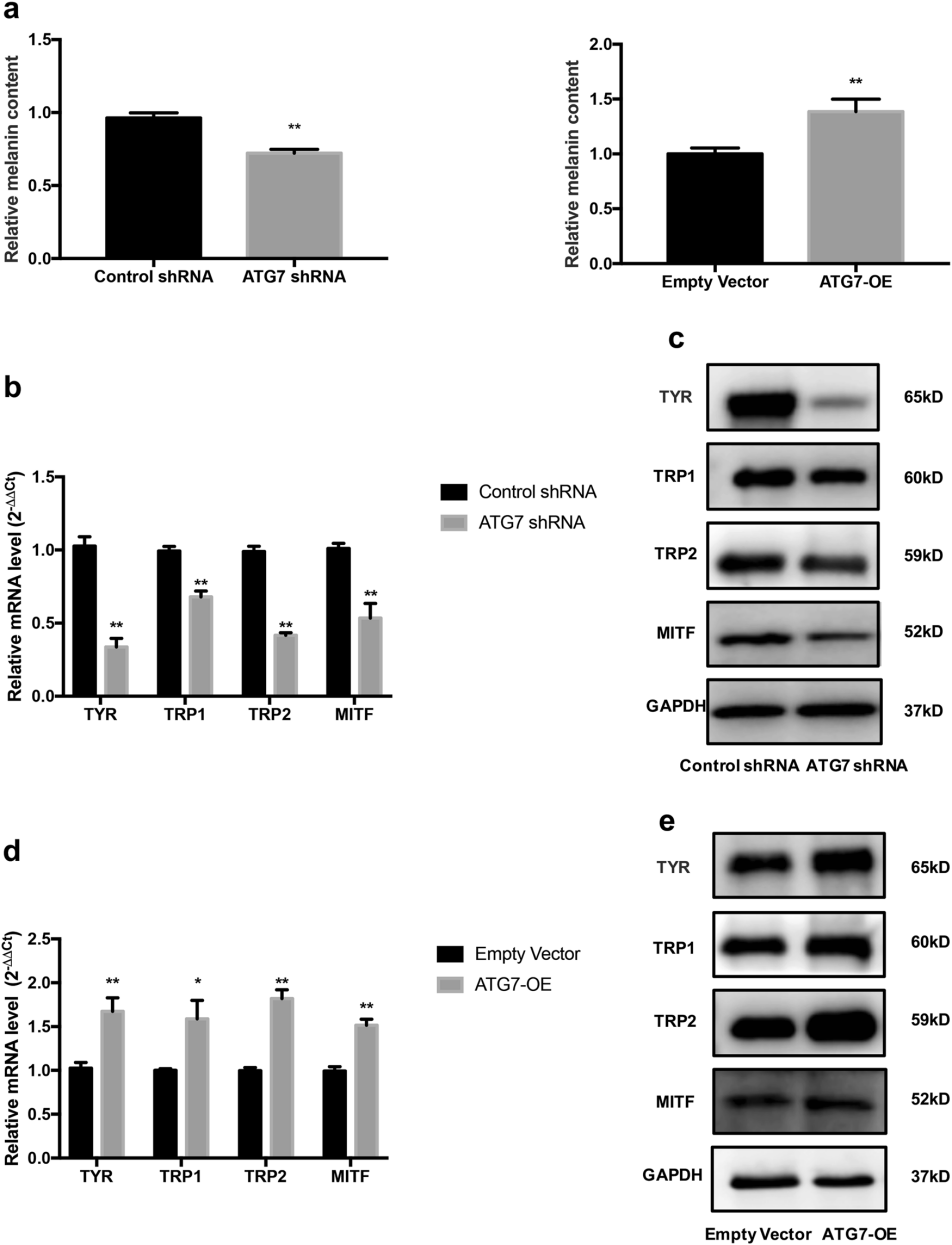
ATG7-dependent autophagy regulates melanin content and melanogenesis of NHEM. **a** Melanin contents of NHEM transfected with control shRNA, ATG7 shRNA, empty vector, or lentivirus ATG7. **b** Relative mRNA expression of TYR, TRP1, TRP2, and MITF in NHEM transfected with control shRNA or ATG7 shRNA were detected by qRT-PCR. **c** Representative images of western blotting of TYR, TRP1, TRP2, and MITF in NHEM transfected with control shRNA or ATG7 shRNA. GAPDH was used as a protein loading control. **d** Relative mRNA expression of TYR, TRP1, TRP2, and MITF in NHEM transfected with empty vector or lentivirus ATG7 were detected by qRT-PCR. **e** Representative images of western blotting of TYR, TRP1, TRP2, and MITF in NHEM transfected with empty vector or lentivirus ATG7. GAPDH was used as a protein loading control. The data represent the mean ± SD from three independent experiments. *P < 0.05, **P < 0.01; ns, nonsignificant.

To investigate whether autophagy was involved in melanin synthesis of NHEM, melanin content and expressions of some crucial genes for melanogenesis, such as tyrosinase (TYR), TYR-related protein 1 (TRP1), TYR-related protein 2 (TRP2), and microphthalmia-associated transcription factor (MITF) were investigated. The relative melanin content of NHEM in ATG7 shRNA group (referred to autophagy-deficient later) was significantly lower than control group (Fig. 2a), whereas the relative melanin content of the ATG7-overexpression group (referred to autophagy-competent later) was significantly higher (Fig. 2a). In addition, the mRNA expressions of TYR, TRP1, TRP2, and MITF were significantly lower in ATG7 shRNA group than control group. The downregulation of TYR, TRP1, TRP2, and MITF was further confirmed by western blotting in NHEM transfected with ATG7 shRNA (Fig. 2b, c). On the contrary, when ATG7 was overexpressed in NHEM, both the mRNA and protein levels of TYR, TRP1, TRP2, and MITF were significantly increased in comparison with those in the empty vector group (Fig. 2d, e). Taken together, these results indicate that ATG7-dependent autophagy is crucial for melanogenesis and it is involved in the process of melanin biosynthesis.

### Deficiency of ATG7-dependent autophagy inhibits proliferation and facilitates oxidative stress-induced apoptosis of NHEM

**Fig. 3:**
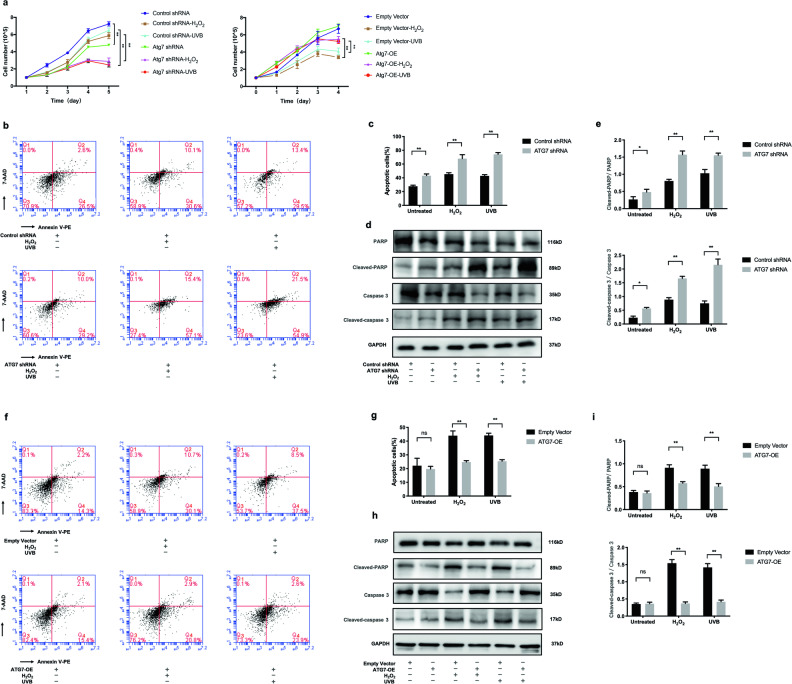
Deficiency of ATG7-dependent autophagy inhibits proliferation and facilitates oxidative stress-induced apoptosis of NHEM. **a** Cell number of NHEM transfected with control shRNA, ATG7 shRNA, empty vector, or lentivirus ATG7, which were treated with or without hydrogen peroxide (H_2_O_2_) and ultraviolet B (UVB) for different durations of time. **b** NHEM transfected with control shRNA or ATG7 shRNA were stimulated with H_2_O_2_ (500 μM) for 24 h or UVB irradiation (12 mW/cm^2^) for 80 s. Levels of apoptosis were detected by flow cytometry assay. **c** Percentage of apoptotic cells in transfected NHEM, which were stimulated with H_2_O_2_ or UVB. **d** Representative images of western blotting of PARP, cleaved PARP, caspase 3, and cleaved caspase 3 in NHEM transfected with control shRNA or ATG7 shRNA, which were stimulated with H_2_O_2_ (500 μM) for 24 h or UVB (12 mW/cm^2^) for 80 s. GAPDH was used as a protein loading control. **e** Statistical analysis of western blotting data of cleaved PARP/PARP and cleaved caspase 3/caspase 3 in NHEM transfected with control shRNA or ATG7 shRNA with or without H_2_O_2_ and UVB. Data are presented as mean ± SD, *n* = 3 in each group. *P < 0.05, **P < 0.01. **f** NHEM transfected with empty vector or lentivirus ATG7 were treated with H_2_O_2_ (500 μM) for 24 h or UVB (12 mW/cm^2^) for 80 s. Levels of apoptosis were detected by flow cytometry assay. **g** Percentage of apoptotic cells in transfected NHEM, which were stimulated with H_2_O_2_ or UVB. **h** Representative images of western blotting of cleaved PARP/PARP and cleaved caspase 3/caspase 3 in NHEM transfected with empty vector or lentivirus ATG7, which were stimulated with H_2_O_2_ (500 μM) for 24 h or UVB (12 mW/cm^2^) for 80 s. GAPDH was used as a protein loading control. **i** Statistical analysis of western blotting data of cleaved PARP/PARP and cleaved caspase 3/caspase 3 in NHEM transfected with empty vector or lentivirus ATG7 with or without H_2_O_2_ and UVB. Data are presented as mean ± SD, *n* = 3 in each group. *P < 0.05, **P < 0.01, ns, nonsignificant.

To explore the role of autophagy in oxidative stress-induced apoptosis, ATG7-transfected NHEM were stimulated with either hydrogen peroxide (H_2_O_2_) or ultraviolet B (UVB) irradiation. The growth curve of NHEM illustrated that suppression of ATG7-dependent autophagy inhibited the proliferation of NHEM not only under normal conditions but upon oxidative stress as well. However, overexpression of ATG7-dependent autophagy only promoted the proliferation of NHEM under oxidative stress (Fig. 3a). To further investigate how ATG7-dependent autophagy inhibited cell proliferation, autophagy-deficient and autophagy-competent NHEM were treated with H_2_O_2_ or UVB irradiation. As shown in Fig. 3b, c, the average percentage of apoptotic cells was readily elevated (43.13 ± 2.53% vs. 27.73 ± 1.52%) in autophagy-deficient NHEM in baseline level and was further elevated (24.64 ± 1.35% vs. 39.76 ± 5.09%) after H_2_O_2_ treatment and (67.83 ± 5.76% vs. 45.27 ± 2.12%) upon UVB irradiation. Moreover, ATG7 knockdown could upregulate the level of cleaved Poly(ADP-ribose)polymerase1(PARP), a nuclear enzyme involved in DNA repair, and cleaved caspase 3 (Fig. 3d, e). By contrast, when autophagy-competent NHEM were treated with H_2_O_2_ or UVB irradiation, the average percentage of apoptotic cells was significantly reduced (24.77 ± 1.00% vs. 43.97 ± 3.49% and 25.37 ± 1.10% vs. 44.10 ± 1.65%) (Fig. 3f, g), in combination with decreased level of cleaved PARP and cleaved caspase 3 (Fig. 3h, i). These results indicate that deficiency of ATG7-dependent autophagy could facilitate oxidative stress-induced apoptosis.

### Deficiency of ATG7-dependent autophagy results in oxidative stress-induced premature senescesnce of NHEM

**Fig. 4:**
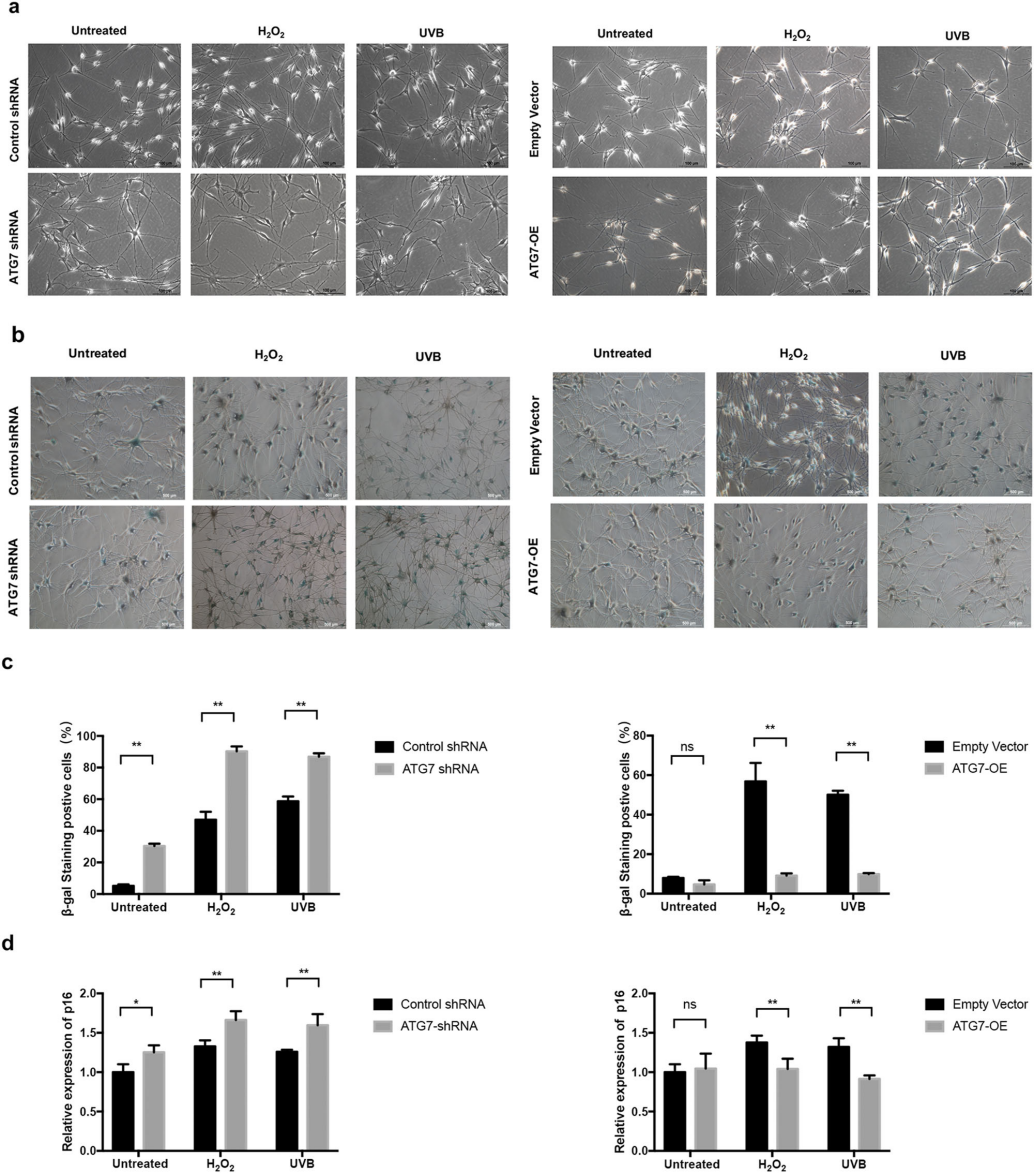
Suppression of ATG7-dependent autophagy leads to premature senescence of NHEM under oxidative stress. **a** Representative images of NHEM, transfected with control shRNA, ATG7 shRNA, empty vector, or lentivirus ATG7 at the third passage, which were treated with or without H_2_O_2_ or UVB. Scale bar = 100 μm. **b** Senescence-associated β-galactosidase (SA β-Gal) staining of NHEM transfected with control shRNA, ATG7 shRNA, empty vector, or lentivirus ATG7, which were treated with or without H_2_O_2_ or UVB. Scale bar = 500 μm. **c** Percentage of SA β-Gal-positive cells in NHEM transfected with control shRNA, ATG7 shRNA, empty vector, or lentivirus ATG7 treated with or without H_2_O_2_ or UVB. **d** Relative mRNA expression of p16 in NHEM transfected with control shRNA, ATG7 shRNA, empty vector, or lentivirus ATG7. Percentage SD from three independent experiments. *P < 0.05, **P < 0.01, ns, nonsignificant.

Next, we characterized the impact of ATG7-dependent autophagy on the aging of NHEM. Autophagy-deficient NHEM started to change morphology early in the third passage and acquired senescent morphotypes with distended cytoplasms. The premature senescent phenotype became even more prominent with the treatment of H_2_O_2_ and UVB irradiation compared with control group. By contrast, no morphology change was observed in autophagy-competent NHEM in baseline condition or under oxidative stress (Fig. 4a). Subsequently, we analyzed two important senescence markers in the transfected NHEM. The activity of senescence-associated β-galactosidase (SA β-Gal) was dramatically increased in autophagy-deficient NHEM in baseline condition or under oxidative stress, whereas the SA β-Gal activity was dramatically decreased in autophagy-competent NHEM only under the treatment of H_2_O_2_ or UVB irradiation (Fig. 4b, c). Moreover, knockdown of ATG7 significantly increased the mRNA level of p16, whereas overexpression of ATG7 could decrease the mRNA expression of p16 in NHEM under the treatment of H_2_O_2_ or UVB irradiation (Fig. 4d). These results suggest that impairment of ATG7-dependent autophagy leads to premature senescence of NHEM. Autophagy may protect NHEM from oxidative stress-induced premature senescence.

### ATG7-dependent autophagy protects NHEM from ROS generation, while deficiency of ATG7-dependent autophagy is responsible for activation of the Nrf2-ARE signaling pathway in NHEM under oxidative stress

**Fig. 5:**
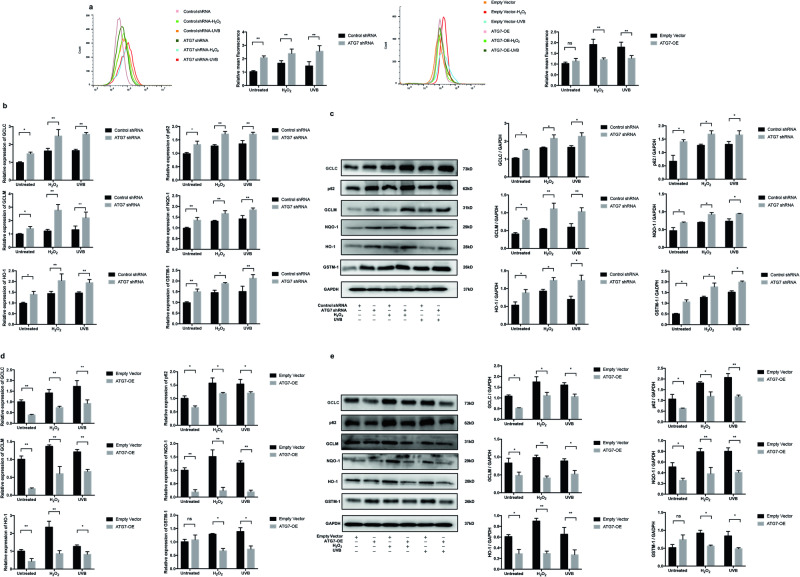
Deficiency of ATG7-dependent autophagy promotes reactive oxygen species (ROS) generation and activation of the Nrf2-ARE signaling pathway in NHEM under oxidative stress, whereas enhancement of autophagy protects NHEM against oxidative stress. **a** NHEM transfected with control shRNA, ATG7 shRNA, empty vector, or lentivirus ATG7 were treated with H_2_O_2_ (200 μM) for 24 h or UVB irradiation (12 mW/cm^2^) for 60 s. Intracellular reactive oxygen species (ROS) levels were measured by flow cytometry assay. Relative quantification of intracellular ROS levels was analyzed in transfected NHEM treated with or without H_2_O_2_ or UVB. **b** Relative mRNA expression of GCLC, p62, GCLM, NQO-1, HO-1, and GSTM-1 in NHEM transfected with control shRNA or ATG7 shRNA. **c** Representative images of western blotting and statistical analysis of western blotting data of GCLC, p62, GCLM, NQO-1, HO-1, and GSTM-1 in NHEM transfected with control shRNA or ATG7 shRNA. GAPDH was used as a protein loading control. Data are presented as mean ± SD, *n* = 3. *P < 0.05, **P < 0.01. **d** Relative mRNA expression of GCLC, p62, GCLM, NQO-1, HO-1, and GSTM-1 in NHEM transfected with empty vector or lentivirus ATG7. **e** Representative images of western blotting and statistical analysis of western blotting data of GCLC, p62, GCLM, NQO-1, HO-1, and GSTM-1 in NHEM transfected with empty vector or lentivirus ATG7. GAPDH was used as a protein loading control. Data are presented as mean ± SD, *n* = 3. *P < 0.05, **P < 0.01, ns, nonsignificant.

We next identified the regulatory role of ATG7-dependent autophagy in ROS production in NHEM. First, autophagy-deficient NHEM were treated with H_2_O_2_ or UVB irradiation. The results showed that knockdown of ATG7-dependent autophagy could accelerate the generation of excessive ROS induced by H_2_O_2_ and UVB treatment. Meanwhile, we performed the same treatment on autophagy-competent NHEM. The results showed that the ROS induced by H_2_O_2_ and UVB irradiation was significantly decreased in the autophagy-competent NHEM compared with the control (Fig. 5a). Taken together, the above data revealed that ATG7-dependent autophagy plays a critical role in protecting NHEM from oxidative stress-induced ROS production.

In autophagy-deficient cells, an accumulation of the adapter protein p62 can be observed frequently. However, p62 is not only the adapter between cargo and the autophagic machinery but also a component of the Nrf2 (nuclear factor (erythroid-derived 2)-like 2)/ARE (antioxidant responsive element) signaling pathway, which is the antioxidant response that provides cellular antioxidants and detoxifying enzymes. Subsequently, we investigated whether the Nrf2 target genes were altered in autophagy-deficient NHEM. Indeed, qRT-PCR analysis showed that the canonical Nrf2 target genes, including γ-glutamyl cysteine ligase (GCLC), SQSTM1/p62, glutamyl cysteine ligase modulatory subunit (GCLM), NAD(P)H dehydrogenase, quinone 1 (NQO-1), heme oxygenase-1 (HO-1), and glutathione *S*-transferase Mu 1 (GSTM-1) were expressed at significantly higher levels in autophagy-deficient NHEM, both under normal conditions and oxidative stress (Fig. 5b). Western blotting analysis further confirmed that the protein levels of GCLC, p62, GCLM, NQO-1, HO-1, and GSTM-1 were increased both under normal conditions and oxidative stress (Fig. 5c). By contrast, the mRNA and protein levels of GCLC, p62, GCLM, NQO-1, HO-1, and GSTM-1 were significantly lower in autophagy-competent NHEM, both under normal condition and oxidative stress (Fig. 5d, e). These data indicate that the dysfunction of ATG7-dependent autophagy results in increased ROS production in NHEM. Autophagy is responsible for the regulation of the Nrf2-ARE signaling pathway under oxidative stress in NHEM.

### ATG7-dependent autophagy causes depletion of antioxidant defense components under oxidative stress

**Fig. 6:**
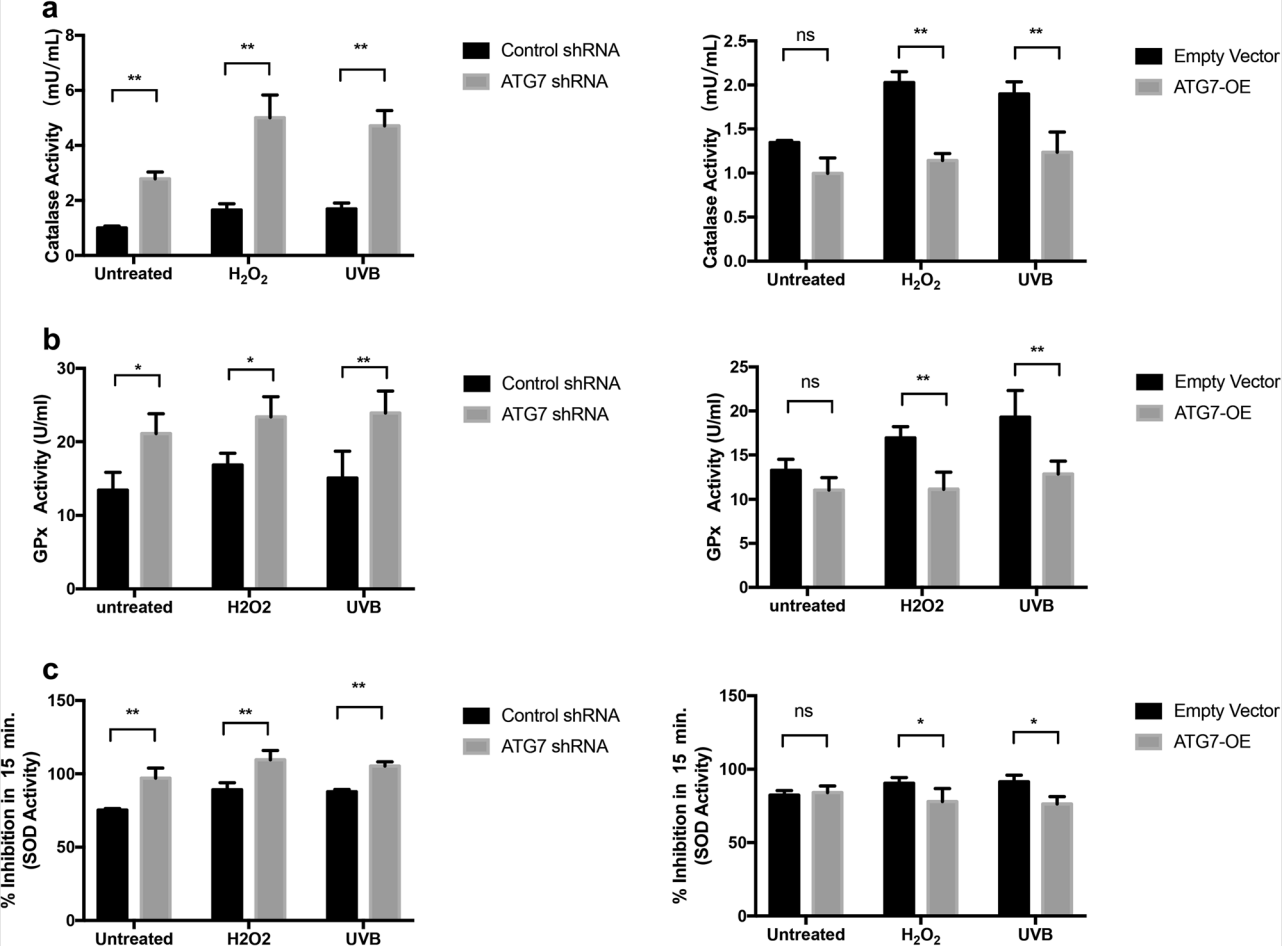
Changes in the activity of antioxidant enzymes in NHEM transfected with control shRNA, ATG7 shRNA, empty vector, or lentivirus ATG7 with or without H_2_O_2_ and UVB treatment. **a** The enzyme catalase (CAT) activity of transfected NHEM treated with or without H_2_O_2_ or UVB. **b** The glutathione peroxidase (GSH-PX) activity of transfected NHEM treated with or without H_2_O_2_ or UVB. **c** The enzyme superoxide dismutase (SOD) activity of transfected NHEM treated with or without H_2_O_2_ or UVB. The data represent the mean ± SD from three independent experiments. *P < 0.05, **P < 0.01, ns, nonsignificant.

Finally, we tried to investigate whether ATG7-dependent autophagy could reduce cytotoxicity by promoting antioxidant defense components under oxidative stress. We revealed that the catalase (CAT) activity was increased in autophagy-deficient NHEM, both under normal condition and oxidative stress (Fig. 6a). However, the CAT activity had no difference between the ATG7-overexpression group and the empty vector group under normal conditions. Only under oxidative stress such as H_2_O_2_ stimulation or UVB irradiation, the CAT activity in autophagy-competent NHEM was significantly decreased compared with control group. In addition, the glutathione peroxidase (GPx) and superoxide dismutase (SOD) activity were significantly increased in autophagy-deficient NHEM, both under normal condition and oxidative stress (Fig. 6b, c). However, the GPx and SOD activity had no significant difference between autophagy-competent NHEM and control group under normal condition. Only under oxidative stress such as H_2_O_2_ stimulation or UVB irradiation, the GPx and SOD activity in autophagy-competent NHEM were significantly decreased (Fig. 6b, c). All the above data demonstrate that ATG7-dependent autophagy is involved in the balance between oxidative stress and antioxidant defense system.

## Discussion

In this study, we investigated the role of ATG7-dependent autophagy in the biological function and oxidative stress responses of primary human melanocytes under oxidative stress.

As is known to all, the primary role of melanocytes is melanin biosynthesis. The destruction of melanocytes is a crucial event in depigmentation skin disorders, such as vitiligo^16,17^. Autophagy has been hypothesized to participate in melanogenesis^18,19^. Here we knocked down ATG7, the central regulator of autophagy, in NHEM and investigated its role in melanogenesis. Our results demonstrated that the genetic knockdown of ATG7-dependent autophagy in NHEM resulted in the reduction of melanin content and dysfunction of melanogenesis due to its suppression on TYR, TRP1, TRP2, and MITF, whereas the genetic overexpression of ATG7-dependent autophagy achieved the opposite results. In that case, alteration of ATG7-dependent autophagy might be involved in the pathogenesis of pigmentation diseases owing to its impact on melanogenesis.

In addition to melanogenesis, the decreased proliferative capacity and apoptosis of melanocytes are crucial in oxidative stress-induced depigmentation. Former studies have confirmed that apoptosis of melanocytes is common in patients with unstable vitiligo, which could be induced by melanocyte detachment and low adhesion^20^. Our study has demonstrated that autophagy-deficient melanocytes differed considerably from normal melanocytes in their proliferative capacity. Suppression of ATG7-dependent autophagy significantly reduces the cell growth rate of melanocytes. In addition, impairment of autophagy renders melanocytes to oxidative stress-induced apoptosis. On the contrary, overexpression of ATG7-dependent autophagy efficiently maintains the proliferative capacity of melanocytes and reduces oxidative stress-induced apoptosis. These findings are in line with earlier reports on lung cancer cells that autophagy suppression may result in inhibition of proliferation and sensitize them to cell apoptosis^21^.

Skin cells are directly exposed to active oxygen species in the environment and are vulnerable to oxidative stress-induced senescence, which results in abnormal aging. Previous studies have revealed that oxidative stress can increase the production of ROS, which in turn aggravates the oxidative damage to the mitochondria^22^. Our study aims to investigate whether autophagy is responsible for protecting NHEM from oxidative stress-induced senescence. Our results indicated that autophagy-deficient melanocytes acquired senescent phenotype much earlier than normal control and lost the ability to fight against premature senescence under oxidative stress, which was consistent with earlier studies^23^. However, the specific molecular mechanisms regulating autophagy-dependent premature senescence under oxidative stress were still unclear. One study has reported the critical role of the SIRT3/AMPK signaling pathway in autophagy activation in the presence of oxidative stress in macrophages^24^, but further studies in melanocytes are still required.

Keap1/Nrf2/ARE signaling pathway is proved to be a critical antioxidant response in protecting melanocytes from oxidative damage^25^. Previous investigations on vitiligo patients have demonstrated that transcript levels of Nrf2 and the downstream detoxification genes such as *GCLC*, *p62*, *GCLM*, *NQO-1*, *HO-1*, and *GSTM-1* were upregulated to protect melanocytes against H_2_O_2_-induced toxicity in vitiligo lesions compared with the matched non-lesional skin^26,27^. Recently, several evidence also support the connection between autophagy and Nrf2/Keap1 signaling pathway by protein p62^28^, but the underlying mechanisms are still not fully understood. During autophagy, intracellular proteins and organelles can be banded to autophagosomes by the adaptor protein p62; meanwhile, the *p62/SQSTM1* is a target gene of Nrf2/Keap1 and creates a positive feedback loop between autophagy and Nrf2/Keap1^29,30^. Komatsu et al.^31^ also found that the accumulation of p62 in autophagy-knockout mice lead to overactivation of the Nrf2 pathway in liver cells and maintaining the stability of autophagy state plays an important role in protecting liver cells from damage stimulated by oxidative stress. On the other hand, the impairment of the Nrf2/Keap1 pathway can lead to defects of autophagy in vitiligo melanocytes^32^. Thus, we can summarize that the impairment of the Nrf2/Keap1 pathway induces the suppression of autophagy while autophagy deficiency leads to the overexpression of the Nrf2/Keap1 pathway. In accordance with the above statements, our study demonstrated that suppression of autophagy enhances activation of the Nrf2/Keap1 signaling and attenuates the ability of NHEM to remove oxidative stress-induced ROS production. These findings strengthen the importance of ATG7-dependent autophagy in melanocytes homeostasis under oxidative stress, providing a potential target for treating depigmentation diseases such as vitiligo.

SOD, GPx, and CAT are important antioxidation enzymes for limiting ROS release and maintaining the integrity of cellular membrane construction^33^. In this study, we assayed the changes in SOD, GPx, and CAT activity. Autophagy deficiency could generate toxicity to cells and promote the activity of CAT, SOD, and GPx so as to decrease ROS accumulation. However, ROS might not be scavenged by elevated CAT, SOD, and GPx instantly and effectively, yet ended up with a significant elevation. Meanwhile, the levels of SOD, CAT, and GPx activity are inconsistent with previous in vivo studies on vitiligo^34–36^, which indicates that the activity of antioxidation enzymes is changing dynamically at different stages of vitiligo. Thus, these results implied that ATG7-dependent autophagy could influence the activity of antioxidation enzymes, but specific mechanisms of how autophagy regulates antioxidation enzymes need further investigation.

In conclusion, our study has uncovered the essential role of ATG7-dependant autophagy in maintaining the normal biological functions of human melanocytes under oxidative stress. Meanwhile, ATG7-dependant autophagy is involved in oxidative stress homeostasis by regulating ROS production, Nrf2 antioxidant signaling pathway, and the activity of several antioxidant enzymes.

To some extent, our findings have proposed a new mechanism for depigmentation skin disorders, that autophagy is indispensable for redox homeostasis and plays a crucial role in oxidative stress-induced melanocyte destruction. Further studies using appropriate depigmented animal models are required to fully clarify the role of autophagy in vivo. Besides, further identification of specific autophagy biomarkers in vitiligo patients might help find a new therapeutic target for vitiligo.

## Materials and methods

### Cell culture

Primary normal human melanocytes were isolated from human foreskin specimens obtained during circumcision surgery. The primary melanocytes were grown in Medium 254 (Cascade Biologics, Portland, OR, USA) containing Human Melanocyte Growth Supplement (Cascade Biologics). The primary melanocytes were used between the second and fourth passages in all experiments. Cells were maintained at 37 °C in a humidified atmosphere containing 5% CO_2_. All subjects consented by written and informed agreement for inclusion in this study. All experiment protocols were approved by the Ethics Committee of the Department of Dermatology, Huashan Hospital, Fudan University.

### Lentiviral infection

Cells were seeded at 2 × 10^5^ cells per well for 24 h before transfection. Cells were infected with ATG7 shRNA (5′-TTTGGGATTTGACACATTT-3′, Genepharma, Shanghai, China) or ATG-OE (5′- AGGTCAAAGGACGAAGATAAC-3′, Genepharma, Shanghai, China) at a 1 : 4 dilution in the presence of 5 μg/mL polybrene (Genepharma, Shanghai, China). The expression of the constructs was confirmed by qRT-PCR and western blotting.

### Quantitative real-time PCR

Total cellular and tissue RNAs were isolated by the Trizol reagent (Invitrogen, Grand Island, NY) and quantified. mRNA expression was tested by using the SYBR GREEN PCR Master Mix (Applied Biosystems, Beijing, China) under a 7500 fast Real-time PCR System (Applied Biosystems). Relative expression of targeted mRNAs was calculated by the 2^−ΔΔCt^ method, using β-actin as the internal control. All the primers were listed in Table 1.

**Table 1:** Sequences of primers for the quantitative RT-PCR (human).

	Name	Forward	Reverse
Human	p62	5′-AGT CGG ATA ACT GTT CAG GAG-3′	5′-ATT CTG GCA TCT GTA GGG A-3′
Human	GCLC	5′-GTG GAT GTG GAC ACC AGA T-3′	5′-GTC TTG CTT GTA GTC AGG AT-3′
Human	GCLM	5′-TGT ATC AGT GGG CAC AGG TA-3′	5′-CAG TCA AAT CTG GTG GCA TC-3′
Human	NQO-1	5′-GAG ACA GCC TCT TAC TTG CC-3′	5′-AAA CCA CCA GTG CCA GTC A-3′
Human	Ho-1	5′-ATT CTC TTG GCT GGC TTC CT-3′	5′-CCT GGA TGT GCT TTT CGT TG-3′
Human	GSTM-1	5′-GGG GAC GCT CCT GAT TAT GA-3′	5′-CGG GCA ATG TAG CAC AAG A-3′
Human	β-Actin	5′-AAG GTG ACA GCA GTC GGT T-3′	5′-TGT GTG GAC TTG GGA GAG G-3′
Human	ATG7	5′-CTG CCA GCT CGC TTA ACA TTG-3′	5′-CTT GTT GAG GAG TAC AGG GTT TT-3′
Human	GAPDH	5′-AGA AGG CTG GGG CTC ATT TG-3′	5′-AGG GGC CAT CCA CAG TCT TC-3′
Human	p16	5′-GAT TGA AAG AAC CAG AGA GGC-3′	5′-GAC CTT CGG TGA CTG ATG AT-3′
Human	TRP1	5′-TGG CAA AGC GCA CAA CTC ACC C-3′	5′-AGT GCA ACC AGT AAC AAA GCG CC-3′
Human	TRP2	5′-TGG GAA ACT GTC TGT GAT AGC-3′	5′-CCA TTT GAT TTC TTC TCA GCA-3′
Human	MITF	5′-ATG CTG GAA ATG CTA GAA TAT AAT-3′	5′-ATC ATC CAT CTG CAT ACA G-3′
Human	TYR	5′-TGG CAT AGA CTC TTC TTG TTG CGG-3′	5′-CAA GGA GCC ATG ACC AGA TCC G-3′

### Western blotting

Equivalent amounts of total cellular lysates (20 μg per treatment) were separated by 10% of SDS-polyacrylmide gel electrophoresis, then transferred to the polyvinylidene fluoride blots (Merck Millipore, Darmstadt, Germany). After blocking in 10% non-fat milk, the blots were incubated with the applied primary antibodies: anti- Atg7 (Cell Signaling, 8558), SQSTM1/p62 (Cell Signaling, 8025), LC3A/B (Cell Signaling, 12741), GAPDH (Cell Signaling, 5174), TRP1 (Abcam, ab178676), TRP2/DCT (Abcam, ab221144), TYR (Abcam, ab170905), MITF (Abcam, ab140606), PARP (Cell Signaling, 9532), cleaved PARP (Cell Signaling, 5625), caspase 3 (Cell Signaling, 14220), cleaved caspase 3 (Cell Signaling, 9661), GCLC (Abcam, ab190685), GCLM antibody (Abcam, ab126704), NQO-1 (Cell Signaling, 62262), HO-1 (Cell Signaling, 5853), or GSTM-1/2/4/5 (Abcam, ab178684) antibodies, followed by incubation with anti-rabbit IgG, horseradish peroxidase-linked secondary antibody (Cell Signaling, 7074). Antibody–antigen binding was detected by an enhanced chemiluminescence substrate kit (Invitrogen), with the results quantified by an ImageJ software (NIH, Bethesda, MD).

### Detection of apoptosis

After experimental treatment, cells were detected by the Annexin V PE/7-AAD Apoptosis Detection Kit (BD Pharmingen, San Diego, CA) following the manufacturer’s instructions and cell apoptosis was analyzed by a flow cytometry machine (Beckman Coulter, Brea, CA).

### SA β-galactosidase staining assay

To measure cellular senescence, cells were stained with the SA β-Gal staining kit (Beyotime Institute of Biotechnology, Nanjing, China) according to the manufacturer’s instructions. Images containing >200 cells were taken using a bright-field microscopy, and total and blue-colored cells were counted. The percentage of SA β-Gal-positive cells was represented by the ratio between the number of blue-colored cells and the number of total cells from at least three independent experiments.

### Assay for reactive oxygen species levels

Melanocytes were seeded at a density of 3 × 10^5^/well on a six-well plate at 37 °C with 5% CO_2_ for 24 h. After H_2_O_2_ and UVB treatment, cells were washed twice with phosphate-buffered saline and detected by the Fluormetric Intracellular Ros Kit (BD Pharmingen, San Diego, CA). Then fluorescence was measured by flow cytometry (Beckman Coulter) within an hour. Mean fluorescence intensity was quantized with Flowjo software (Treestar USA).

### Statistical analysis

Each experiment was performed at least three times and statistical analyses of the data were performed using unpaired, two-tailed Student’s *t*-tests or using a two-way analysis of variance, followed by Newman–Keuls test built into GraphPad Prism (GraphPad Software 5.0; San Diego, CA). All data were expressed as mean ± SD. *P* < 0.05 were considered statistically significant.
