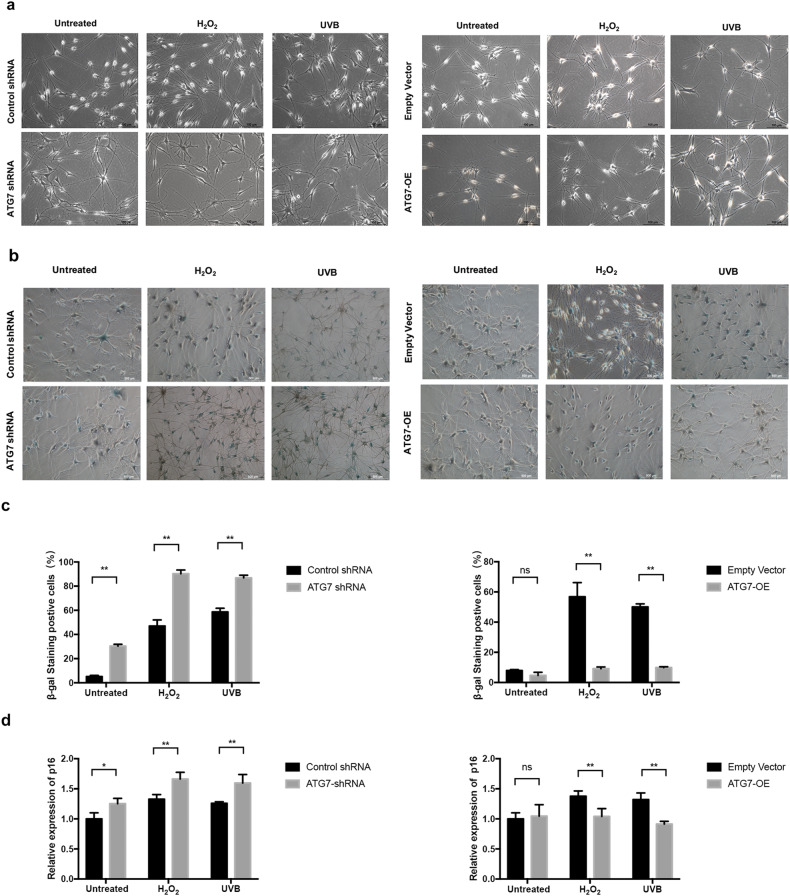# Correction: Dysfunction of ATG7-dependent autophagy dysregulates the antioxidant response and contributes to oxidative stress-induced biological impairments in human epidermal melanocytes

**DOI:** 10.1038/s41420-024-02050-y

**Published:** 2024-07-03

**Authors:** Zhuhui Qiao, Zhongyi Xu, Qing Xiao, Yiwen Yang, Jiayi Ying, Leihong Xiang, Chengfeng Zhang

**Affiliations:** Department of Dermatology, Huashan Hospital, Fudan University, Shanghai, China

Correction to: *Cell Death Discovery* 10.1038/s41420-020-0266-3, published online 01 May 2020

In Fig. 4a, one image (H_2_O_2_, ATG7 shRNA) was mistakenly uploaded and duplicated with another one (UVB, Control shRNA). We apologize for the mistakes that the image (UVB, Control shRNA) was shown twice in Fig. 4a, while the original image (H_2_O_2_, ATG7 shRNA) was not shown. This correction does not affect the remainder of the article and will not affect the conclusion of the article.

The original article has been corrected.

Incorrect Fig. 4:
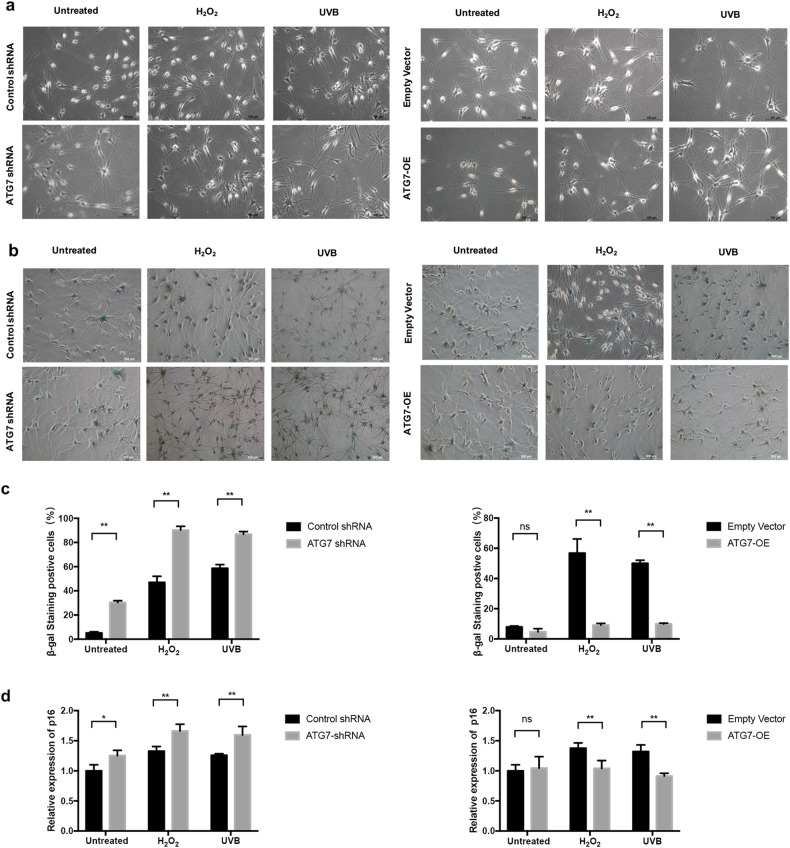


Corrected Fig. 4: